# Comparison of a Kidney Replacement Therapy Risk Score Developed in Kaiser Permanente Northwest vs Estimated Glomerular Filtration Rate in Advanced Chronic Kidney Disease Using Decision Curve Analysis

**DOI:** 10.7812/TPP/21.104

**Published:** 2021-12-07

**Authors:** Ken J Park, Jose G Benuzillo, Erin Keast, Micah L Thorp, David M Mosen, Eric S Johnson

**Affiliations:** ^1^ Department of Nephrology, Kaiser Permanente Northwest, Portland, OR; ^2^ Edwards Lifesciences, Irvine, CA; ^3^ Kaiser Permanente Center for Health Research Northwest, Portland, OR; ^4^ Department of Analytics, Kaiser Permanente Northwest, Portland, OR

**Keywords:** arteriovenous fistula, chronic kidney disease, prediction, prognosis, risk score

## Abstract

**Introduction:**

Use of kidney replacement therapy (KRT) prediction models for guiding arteriovenous fistula (AVF) referrals in advanced chronic kidney disease (CKD) is unknown. We aimed to compare a hypothetical approach using a KRT prediction model developed in Kaiser Permanente Northwest to estimated glomerular filtration rate (eGFR) for AVF referrals.

**Methods:**

Our retrospective cohort consisted of patients with stage G4 CKD in Kaiser Permanente Northwest followed by nephrology. Two-year KRT risk was calculated at each nephrology visit up to 2 years from entrance into cohort based on a previously published model. We calculated sensitivity, specificity, and area under the receiver operating characteristic curve (AUC) based on several 2-year KRT risk and eGFR cutoffs for outcome of hemodialysis at 18 months. We compared an approach of AVF referral using 2-year KRT risk and eGFR cutoffs using decision curve analysis.

**Results:**

Two-year KRT risk better discriminated progression to hemodialysis compared to eGFR < 15 mL/min (AUC 0.60 vs 0.69 at 2-year KRT risk > 20% and 0.69 at 2-year KRT risk > 40%, p = 0.003 and 0.006, respectively) but not to eGFR of 20 mL/min (AUC 0.64, p = 0.16 and 0.19, respectively). Decision curve analysis showed that AVF referral guided by 2-year KRT risk score resulted in higher net benefit compared to eGFR at low thresholds for referral.

**Conclusion:**

In stage G4 CKD, a 2-year KRT risk model better predicted progression to KRT at 18 months compared to an eGFR of 15 mL/min but not to 20 mL/min and may improve timely referral for AVF placement in patients at lower thresholds for referral.

## INTRODUCTION

Chronic kidney disease (CKD) is estimated to affect about 10% to 15% of the US population.^1^ While the majority of patients with CKD do not progress to end-stage kidney disease, in those patients at risk, adequate planning is recommended to avoid starting hemodialysis with a central venous catheter (CVC).^2^ CVC use at initial hemodialysis has been associated with increased mortality, increased hospitalizations, sepsis, and higher health care costs.^3^^–^^5^ However, even with pre-dialysis nephrology care, CVC use remains high.^6^

Several challenges contribute to high CVC rates at initial hemodialysis including patient refusal, late referral to nephrology, arteriovenous fistula (AVF) maturation failure, and suboptimal timing of AVF placement.^7^^–^^9^ The development of several models for predicting risk of progression to kidney replacement therapy (KRT) in CKD are promising tools that may help guide the timing of AVF placement.^10^^,^^11^ Recent guidelines by the National Kidney Foundation’s Kidney Disease Outcomes Quality Initiative recommend AVF referral at an estimated glomerular filtration rate (eGFR) of 15-20 mL/min^2^. However, there is little guidance about the use of prediction models. Our study aim was to compare a hypothetical approach using a KRT prediction model developed in Kaiser Permanente Northwest (KPNW) vs eGFR for guiding AVF referral in a retrospective cohort of advanced CKD patients followed by nephrology.

## METHODS

### Study Design, Population, and Setting

Our study population was a retrospective cohort of patients with stage G4 CKD followed by nephrology who were enrolled in KPNW. KPNW is a large integrated health care system serving Oregon and Washington with an annual membership of around 600,000 members staffed by 10 nephrologists. Patients were eligible for the cohort if they were between the ages of 20 to 89 years with prevalent and incident stage G4 CKD (defined as 2 eGFR measurements using the CKD Epidemiology Collaboration equation ≥ 15 and < 30 mL/min occurring 90 to 730 days apart, without an intervening eGFR < 15 or ≥ 30 mL/min) between May 1, 2013 and May 1, 2016. Entrance into the cohort started at the date of the second qualifying eGFR. Patients who had a prior kidney transplant, a history of acute kidney injury requiring hemodialysis, or who had been enrolled for less than 12 months before cohort entry were excluded.

We tabulated eGFR and retrospectively calculated 2-year KRT risk at each nephrology visit up to 24 months from entrance into the cohort. The 2-year KRT risk was calculated based on a prediction model developed in KPNW by Schroeder et al,^11^ which includes age, sex, eGFR, hemoglobin, presence of proteinuria or albuminuria, systolic blood pressure, antihypertensive use, and the Diabetes Complications Severity Index. The diabetes index was based on ICD-9 and -10 codes, which includes retinopathy, neuropathy, cerebrovascular disease, cardiovascular disease, peripheral vascular disease, and metabolic complications such as diabetic ketoacidosis.^12^ Proteinuria or albuminuria was defined as being present if the spot urine albumin creatinine ratio was ≥ 30 mg/g, the spot urine protein creatinine ratio was ≥ 150 mg/g, or the urine dipstick test was ≥ 1+. Antihypertensive use was based on antihypertensive medication fills within 90 days of the visit. Our study cohort was separate from that used to develop the prediction model.

### Statistical Methods and Outcomes

Our outcome of interest was the initiation of hemodialysis. The dates of hemodialysis, transplantation, AVF referral, and initial access used at hemodialysis were obtained through review of electronic health records. Patients were followed for the outcome of hemodialysis up to 42 months from entrance into the cohort. Patients were censored if they changed insurance coverage (3% of patients) or at time of death. Patients were defined as having a positive test if they had a nephrology visit with a 2-year KRT risk > 20% or 40%, or eGFR < 15 or 20 mL/min at any nephrology visit within 2 years from entrance into the cohort. We chose these cutoffs based on expert opinion and Kidney Disease Outcomes Quality Initiative guidelines.^2^^,^^13^ We defined a negative test as patients that did not reach the specified 2-year KRT or eGFR cutoffs at any nephrology visit within 2 years from entrance into the cohort. We calculated the specificity, sensitivity, and area under the receiver operating characteristic curve on the outcome of progression to hemodialysis within 18 months of the first nephrology visit with eGFR < 15 or 20 mL/min, or 2-year KRT risk > 20% or 40%. We excluded patients with an incalculable 2-year KRT risk score, followed less than 6 months by nephrology, started on peritoneal dialysis, or received a preemptive kidney transplant from final analysis. A missing proteinuria measurement was the most common cause for an incalculable risk score. Our initial cohort consisted of 1,075 patients, which decreased to 959 patients after excluding 18 patients with incalculable 2-year KRT risk score, 43 patients followed less than 6 months by nephrology prior to hemodialysis, 46 patients that started on peritoneal dialysis, and 9 patients that received preemptive kidney transplants.

We evaluated the benefit of AVF referral using eGFR or a 2-year KRT risk cutoff using decision curve analysis (DCA). DCA is a method examining the benefit of a prediction model or diagnostic test introduced by Vickers and Elkin.^14^ For our analysis, we looked at the 2-year KRT risk score and eGFR at the first nephrology visit after entrance into the cohort and subsequent nephrology visit 1 year after the first visit. We compared the net benefit for outcome of hemodialysis within 18 months of that visit for an eGFR < 15 or 20 mL/min, or a 2-year KRT risk greater than 20% or 40%. DCA compares the net benefit of a prediction model or diagnostic test by subtracting the expected harm (false positive/total number) multiplied by odds at a threshold from the expected benefit (true positive/total number). The threshold can be adjusted based on the clinician’s and patient’s preference. A low threshold may be appropriate where the harm of missing a diagnosis is high and the harm of the test is low. A high threshold may be appropriate where harm that results from the test is high in those patients that do not develop the outcome. The threshold can also be thought of as the number needed to treat. For example, at a threshold of 10%, the clinician would be willing to treat 10 patients if 1 develops the outcome. Conversely, at a threshold of 50%, the clinician would only be willing to treat 2 patients if 1 develops the outcome. The net benefit in DCA is represented as a curve, which is compared to no treatment and treatment of all patients with the net benefit shown on the y-axis and the thresholds shown on the x-axis. A prediction model that has a higher net benefit compared to another test or prediction model over a defined range of thresholds would suggest that the clinician should use that prediction model.

The CKD cohort and risk score dataset were built using SAS version 9.4 (SAS Institute Inc, Cary, NC). Statistical analysis was done using Stata 16 statistical software (StataCorp LP, College Station, TX) and R version 3.6.2.^15^ Means were calculated for continuous variables, while proportions were calculated for categorical variables. Statistical significance was defined as p < 0.05. Standard deviations were reported for means. A publicly available code was used to calculate DCA.^16^ This study was reviewed and approved by the institutional review board of KPNW and conducted in accordance with the ethical standards laid down in the Declaration of Helsinki.

## RESULTS

Table 1 shows baseline characteristics at the beginning of the cohort. Patients in the cohort were predominantly elderly with an average age of 74. Table 2 shows that 16% had progressed to hemodialysis and 17% had died. About 12% had an eGFR < 15 mL/min, 39% had an eGFR < 20 mL/min, 44% had a 2-year KRT risk score > 20%, and 23% had a 2-year KRT risk score > 40% at any point within 2 years from entrance into the cohort.

**Table 1.** Characteristics of patients with G4 chronic kidney disease (n = 959) at beginning of cohort

Characteristic	
Mean age, years (± SD)	74 ± 10
Sex (female), %	52
Diabetes, %	63
Mean eGFR (± SD)	24 ± 4
Mean nephrology visits (± SD)	5 ± 3

eGFR = estimated glomerular filtration rate.

**Table 2.** Outcomes of patients with stage G4 chronic kidney disease up to 42 months after entrance into the cohort

Characteristic	No. (%)
AVF placed	142 (15%)
Progressing to hemodialysis	92 (65%)
Not progressing to hemodialysis	50 (35%)
Hemodialysis	152 (16%)
Initial hemodialysis access	
Central venous catheter	55 (36%)
AVF	86 (57%)
AVG	11 (7%)
Death	165 (17%)
eGFR < 15 mL/min	116 (12%)
eGFR < 20 mL/min	375 (39%)
2-year KRT > 20%	418 (43%)
2-year KRT > 40%	220 (23%)

AVF = arteriovenous fistula; AVG = arteriovenous graft; eGFR = estimated glomerular filtration rate; KRT = kidney replacement therapy.

We examined sensitivity and specificity at an eGFR < 15 and 20 mL/min and a 2-year risk KRT > 20% and 40% for the outcome of KRT at 18 months (Table 3). Specificity increased using the lower eGFR while sensitivity decreased. Similarly, specificity increased using the higher 2-year KRT risk score while sensitivity decreased. The area under the receiver operating characteristic curve was significantly greater using 2-year KRT risk > 20% and 40% compared to using eGFR < 15 mL/min (p = 0.003 and 0.006, respectively) but not compared to an eGFR < 20 mL/min (p = 0.16 and 0.19, respectively).

**Table 3.** Tests to identify G4 chronic kidney disease requiring hemodialysis within 18 months

Cutoff	Specificity (95% CI)	Sensitivity (95% CI)	Area under the curve (95% CI)
eGFR < 20 mL/min	0.64 (0.60-0.67)	0.64 (0.54-0.74)	0.64 (0.59-0.69)
eGFR < 15 mL/min	0.91 (0.88-0.92)	0.29 (0.21-0.37)	0.60 (0.56-0.64)
2-year KRT risk > 20%	0.60 (0.56-0.63)	0.78 (0.68-0.87)	0.69 (0.64-0.74)
2-year KRT risk > 40%	0.81 (0.78-0.83)	0.57 (0.46-0.67)	0.69 (0.64-0.74)

CI = confidence interval; eGFR = estimated glomerular filtration rate; KRT = kidney replacement therapy.

Table 4 shows the number of AVF referrals that would have been recommended in our cohort using an eGFR < 15 and 20 mL/min compared to 2-year KRT risk > 20% and 40%. In our cohort, 64 patients were referred for AVF placement greater than 6 months prior to progression to hemodialysis, while 88 patients were not referred or referred < 6 months prior to starting hemodialysis. Using an eGFR < 20 mL/min, or 2-year KRT risk > 20% or 40% would have identified a greater number of patients that would have progressed to hemodialysis but also increased referrals in patients who would not have reached hemodialysis. Using an eGFR < 15 mL/min would have resulted in the lowest number of referrals in patients not progressing to hemodialysis but also the lowest number of referrals in patients that would have reached hemodialysis.

**Table 4.** Number of patients that would have been identified correctly and identified incorrectly progressing to hemodialysis within 42 months using different eGFR and 2-yr KRT cutoffs

	Observed	eGFR < 20 mL/min	eGFR < 15 mL//min	2-year KRT > 20%	2-year KRT > 40%
Percent of unnecessary referral in patients not progressing to hemodialysis	9% (75/807)	36% (266/807)	9% (70/807)	40% (289/807)	19% (118/807)
Percent of total patients progressing to hemodialysis identified	42% (64/152^a^)	64% (109/152)	29% (46/152)	78% (129/152)	57% (102/152)

a Excludes patients referred for arteriovenous fistula < 6 months before starting hemodialysis.

eGFR = estimated glomerular filtration rate; KRT = kidney replacement therapy.

**Figure 1.**
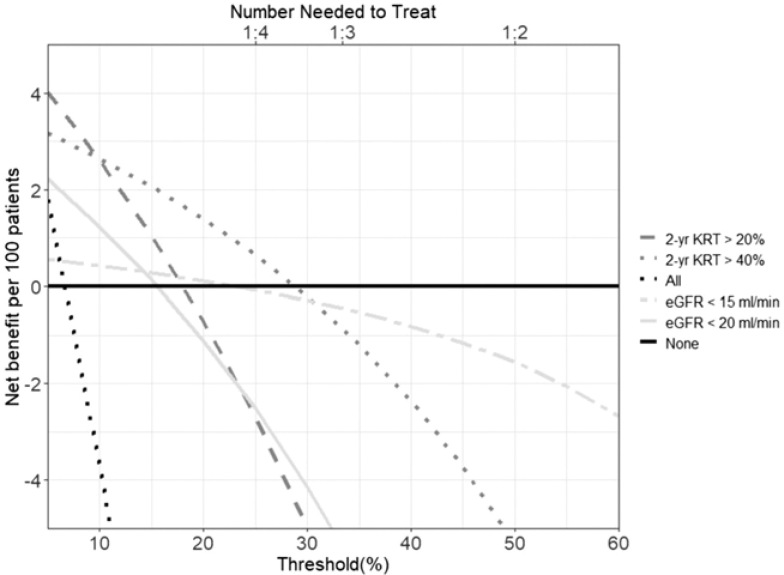
Decision curve analysis illustrating net benefit using 2-year kidney replacement therapy risk score > 20% and 40% and estimated glomerular filtration rate < 15 and 20 mL/min compared to referral of no patients and referral of all patients for arteriovenous fistula placement. eGFR = estimated glomerular filtration rate; KRT = kidney replacement therapy.

DCA showed that referral based on 2-year KRT risk > 40% provided the highest net benefit compared to referral based on eGFR and referral of all and no patients up to a threshold risk of 28% (Figure 1). Above a threshold of 28%, AVF referral based on an eGFR < 15 mL/min resulted in a higher net benefit compared to a 2-year KRT risk score of 20% or 40% or an eGFR < 20 mL/min but no net benefit compared to referral of no patients.

## DISCUSSION

Our findings demonstrate that use of a KRT risk score may help augment the nephrologist’s clinical intuition in AVF planning in patients with stage G4 CKD. Our study suggests that a 2-year KRT risk score better discriminated patients’ progression to KRT at 18 months compared to eGFR < 15 mL/min but not to an eGFR < 20 mlL/min. In addition, DCA shows that at lower thresholds for AVF referral, a 2-year KRT risk score > 40% resulted in the highest net benefit for timely referral, while at higher thresholds for AVF referral, eGFR < 15 mL/min resulted in the highest net benefit.

Current guidelines for AVF referrals rely on projection of progression to hemodialysis or eGFR cutoffs. These guidelines try to balance the harms of late AVF placement, which increases the likelihood of starting hemodialysis with a CVC with too early AVF placement, which results in increased interventions to maintain patency.^8^^,^^17^ Using a simulation study, Shechter et al^18^ showed that AVF referral at 12-18 months before dialysis or at an eGFR of 15-20 mL/min was associated with optimal balance between patients starting hemodialysis with an AVF and being referred for unnecessary AVF placement. However, using eGFR to optimally time AVF referrals remains difficult due to differing rates in eGFR decline and higher risk for death in older patients.^19^^,^^20^ Despite these limitations, Kidney Disease Outcomes Quality Initiative recommends referral at an eGFR of 15-20 mL/min^2^.

Several prediction models have been published predicting KRT risk in patients with CKD.^10^^,^^11^^,^^21^ These models show good discrimination and perform as well if not better compared to nephrologists’ intuition in predicting which patients progress to KRT.^22^ Expert opinion has suggested using these prediction models to guide referral to nephrology or KRT planning.^13^ However, few studies have examined their effects in clinical practice. One study found a significant decrease in median wait time to nephrology after instituting a 5-year KRT risk > 3% based on the Kidney Failure Risk Equation (KFRE) as part of the criteria for referral.^23^

Our study provides evidence that the use of a KRT risk model may augment the nephrologist’s decision of when to refer for AVF placement. For example, in a 45-year-old man with multiple complications from diabetes, high-grade proteinuria with an eGFR of 22 mL/min, and a 2-year KRT risk > 40%, the clinician may have a lower threshold for referring this patient for AVF placement. DCA suggests that this patient would more likely benefit from earlier referral, rather than waiting to an eGFR of 15 mL/min. Conversely, in an 85-year-old woman without diabetes and proteinuria with an eGFR of 20 mL/min and a low 2-year KRT risk, the clinician may have a higher threshold for AVF referral. DCA suggests that this patient would less likely benefit from AVF referral at her current eGFR. Our study findings add to a recent study by Ali et al,^24^ which showed that use of KRFE would result in better clinical utility compared to eGFR thresholds in advanced CKD.

Our study has several limitations. The KPNW risk score was developed in a CKD population that predominantly had stage G3 CKD and has not been validated in stage G4 CKD.^11^ A recent study found that the KFRE and the 4-year Grams model had the highest calibration and discrimination in patients with advanced CKD.^25^ We were unable to compare other published prediction models, as most of our cohort had urinary protein rather than urinary albumin measurements, and recently published equations converting urine protein to urine albumin were not available at the time of our analysis.^26^^,^^27^

The observational nature of our study limited our ability to assess the impact of applying risk score in increasing timely AVF referrals. Other factors such as late nephrology referrals, hospitalizations, and patient indecision play an important role in delays in AVF referral. In addition, the exclusion of patients who only had one eGFR < 30 mL/min could have resulted in selection bias. A prospective trial applying a KRT prediction model in clinical practice is needed to assess its impact on increasing AVF use at initial hemodialysis.

There are several limitations with using DCA. DCA does not provide a framework for performing a more complex cost effectiveness analysis. Deciding when to refer for AVF placement is complex, as the clinician is trying to balance the benefit with having a patient starting hemodialysis with an AVF to the cost and burden of AVF placement if the patient does not progress to end-stage kidney disease or requires interventions to maintain its patency if placed too early. The net benefit of DCA is also dependent on the prevalence of the outcome of interest. The results of this study may not be applicable to other populations or health systems where prevalence of end-stage kidney disease is higher, as our patient population was predominantly White (∼80%) and in the Pacific Northwest, which has a lower rate of end-stage kidney disease compared to other regions.^28^

## CONCLUSION

In summary, our study suggests that use of a prediction model for KRT may be an important tool for AVF planning in advanced CKD. Future prospective studies are needed to see if application of KRT prediction models in clinical practice improves timeliness of AVF referral.
